# Interplay between APP and glypican-1 processing and α-synuclein aggregation in undifferentiated and differentiated human neural progenitor cells

**DOI:** 10.1093/glycob/cwad013

**Published:** 2023-02-15

**Authors:** Fang Cheng, Lars-Åke Fransson, Katrin Mani

**Affiliations:** Department of Experimental Medical Science, Division of Neuroscience, Glycobiology Group, Lund University, Biomedical Center A13, Lund SE-221 84, Sweden; Department of Experimental Medical Science, Division of Neuroscience, Glycobiology Group, Lund University, Biomedical Center A13, Lund SE-221 84, Sweden; Department of Experimental Medical Science, Division of Neuroscience, Glycobiology Group, Lund University, Biomedical Center A13, Lund SE-221 84, Sweden

**Keywords:** amyloid precursor protein, glypican-1, heparan sulfate, Parkinson’s disease, α-synuclein

## Abstract

In Parkinson’s disease, there is an accumulation of α-synuclein (SYN) aggregates in neurons, which is promoted by neuroinflammation. In neural cells, cytokine-induced SYN aggregation is modulated by heparan sulfate (HS) derived from glypican-1 (GPC1) by amyloid precursor protein (APP) and nitric oxide (NO)-dependent cleavage. We have explored possible interplay between APP, GPC1, and SYN in undifferentiated and differentiated neural progenitor cells (NPCs) by modulating APP and GPC1 processing. Effects were monitored by immunofluorescence microscopy and slot immunoblotting using antibodies recognizing APP degradation products, HS released from GPC1, and SYN aggregates (filamentous SYN [SYNfil]). Suppression of HS release from GPC1 by inhibition of β-secretase or by NO deprivation resulted in no or slight increase in SYNfil aggregation. Stimulation of HS release by ascorbate did not further increase SYNfil staining. Interleukin-6 (IL-6) induced increased APP and GPC1 processing and SYNfil formation, which was reduced when β-secretase was inhibited and when HS release was impeded by NO deprivation. Ascorbate restored APP and GPC1 processing but did not affect SYNfil formation. Ascorbate-dependent differentiation of NPC resulted in the expression of tyrosine hydroxylase (TH) which colocalized with SYNfil. Suppression of APP processing by inhibition of β-secretase greatly disturbed the differentiation process. IL-6 induced coclustering of APP-degradation products, TH, HS, and SYNfil, which could be reversed by stimulation of HS release from GPC1 by excess ascorbate. We suggest that continuous release of HS from GPC1 moderates SYN aggregation and supports differentiation of NPC to dopaminergic neurons.

## Introduction

Alzheimer’s disease (AD) and Parkinson’s disease (PD) are the most prevalent neurodegenerative disorders. Increased processing of the amyloid precursor protein (APP) by β-secretase and γ-secretase, resulting in the accumulation of misfolded, aggregation-prone, and toxic amyloid beta (Aβ) peptides, is considered to be the hallmark of AD (for reviews, see e.g. Benilova et al. 2012; De Strooper and Karran 2016). In PD, there is an accumulation of α-synuclein (SYN) aggregates in dopaminergic neurons (for review, see e.g. Guardia-Laguarta et al. 2015). In many dementia cases, there are also mixed AD-PD neuropathological features, and Aβ and SYN peptides occur together in amyloid plaques (for review, see e.g. Bendor et al. 2013). SYN is widely expressed by many neuronal populations within both central and peripheral systems (see Bendor et al. 2013). Similarly, APP expression and production of Αβ occurs both in hippocampal and cortical neurons as well as in peripheral tissues (for reviews, see Haughey et al. 2002; Morris et al. 2014; Wang et al. 2017). Moreover, in vitro studies have shown that Aβ peptides can trigger aggregation of SYN (Köppen et al. 2020).

**Fig. 1:**
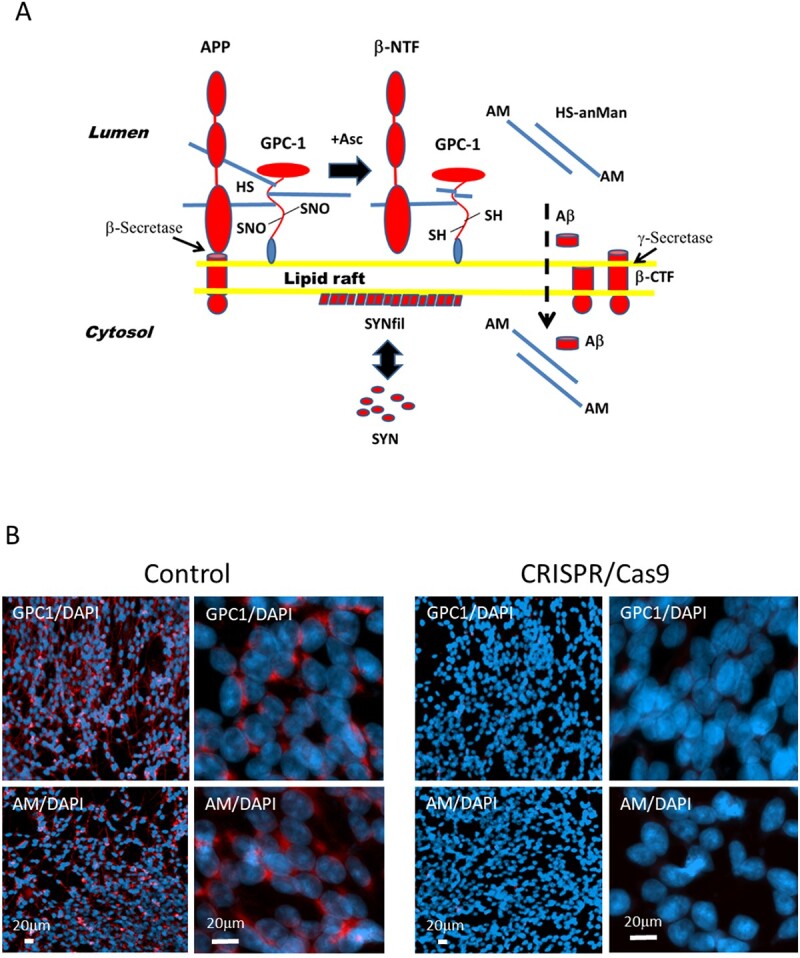
A) Processing of APP and GPC1. anMan/AM, anhydromannose; Asc, ascorbate; β-CTF, C-terminal fragment of APP generated by β-secretase cleavage; β-NTF, N-terminal fragment of APP generated by β-secretase cleavage; SH, thiol; HS is denoted by the blue rod. B) Representative immunofluorescence images at low and high magnifications of neuronal progenitor cells (NPCs) after knockout of *GPC1*. Sparse cultures were transfected with either a nonspecific CRISPR/Cas9 plasmid (control), or a *GPC1*-targeted knockout plasmid (CRISPR/Cas9), and then grown to near confluence. Staining was performed with pAb GPC1 (red) or mAb AM (red) and DAPI (for nuclei, blue). Exposure time was the same in both cases.

APP and the recycling heparan sulfate (HS)-containing proteoglycan glypican-1 (GPC1) bind strongly to one another (Reinhard et al. 2005; Mani et al. 2006). GPC1 becomes S-nitrosylated (S-nitrosothiol [SNO]) during caveolin-/lipid raft-dependent endocytosis (Cheng et al. 2002; Watanabe et al. 2004). In endosomes, GPC1 is attached to the luminal side of lipid rafts via its C-terminal glycosyl-phosphatidyl-inositol anchor (Fig. 1A, middle section; see also Watanabe et al. 2004). APP, which is a type I membrane-spanning protein, can bind to GPC1, both via protein-to-protein and protein-to-HS interactions (Fig. 1A, left side; see also Reinhard et al. 2005). Moreover, APP and GPC1 colocalize in cytoplasmic compartments of neuroblastoma cells (Cappai et al. 2005).

Cleavage of APP by β-secretase releases a soluble N-terminal fragment (β-NTF), while the C-terminal fragment (β-CTF) remains inserted in the membrane (Fig. 1A, right side). This fragment can then be further degraded by γ-secretase, yielding Αβ peptides. GPC1 releases its HS chains by an intrinsic SNO-catalyzed deaminative cleavage generating HS chains and oligosaccharides containing a reducing terminal anhydromannose (HS-anMan/AM). This reaction is initiated by β-NTF and stimulated by ascorbate (Fig. 1A, middle section). HS-anMan penetrates the membrane, possibly facilitated by β-CTF, and is then transported to the nucleus, probably carried by nucleolin. After leaving the nucleus, HS-anMan is captured by autophagosomes and degraded in lysosomes (Mani et al. 2006; Cheng et al., 2011, 2012, 2014; Cheng, Belting, et al. 2017; Cheng, Fransson, et al. 2017).

Aβ is also transported into the cytosol and then to the nucleus (Fig. 1A, right side). Exogenously supplied Aβ peptides and oligomers are taken up by neuroblastoma cells and are transferred to the nucleus (Cheng et al. 2013; Barucker et al. 2014). Also, endogenously generated Aβ display nuclear transit in fibroblasts from the AD mouse Tg2576 (Cheng et al. 2015a). Wild-type mouse fibroblasts exposed to nitric oxide (NO) deprivation or inhibition of lysosome function accumulate Aβ first in the nucleus and then in autophagosomes (Cheng et al. 2015b).

SYN, which is present in the cytosol, can bind to the cytosolic surface of lipid rafts. Binding to these rafts alters the conformation of the N-terminal domain of SYN, which facilitates filament formation (Fig. 1A, filamentous SYN [SYNfil]; see also Guardia-Laguarta et al. 2015).

Proinflammatory cytokines contribute to neurodegeneration in both AD and PD (for reviews, see Heneka et al. 2015; Calsolaro and Edison 2016; Joshi and Singh 2018; Rocha et al. 2018). We have shown that proinflammatory cytokines, such as tumor necrosis factor-alpha (TNF-α), interleukin-1-beta (IL-1β), and interleukin-6 (IL-6) induce the accumulation of GPC1-derived HS-anMan and APP-derived β-CTF and also aggregation of SYN in dividing neuroblastoma cells, neural stem cells, and neural progenitor cells (NPCs), while the nondividing cortical neurons are unaffected (Cheng et al. 2020, 2022).

To compare the effect of proinflammatory cytokines on growing neural stem cells before and during differentiation after growth arrest, we have used human NPCs, which can be differentiated to dopaminergic neurons. We find that the cytokine IL-6 induces an APP-dependent aggregation of SYN both during the growth phase and during the differentiation to tyrosine hydroxylase (TH)-expressing NPC. By supporting a continuous release of HS-anMan from GPC1, the aggregation of SYN can be moderated.

## Results

### Inhibition of APP processing induces coclustering of GPC1-derived HS-anMan and Aβ in NPC

To show that HS-anMan was derived from GPC1, we used CRISPR/Cas9 technology to knock out the GPC1 gene (*GPC1*). NPCs were transfected with a green fluorescent protein-tagged nonspecific vector or a similarly tagged *GPC1*-targeted vector and were then examined by deconvolution immunofluorescence microscopy after staining with antiserum against human GPC1 or monoclonal antibody (mAb) AM to detect HS-anMan. In the control, there was an expression of GPC1 and also formation of HS-anMan, both located in vesicular structures, while knockout of *GPC1* resulted in the complete absence of HS-anMan formation (Fig. 1B).

**Fig. 2:**
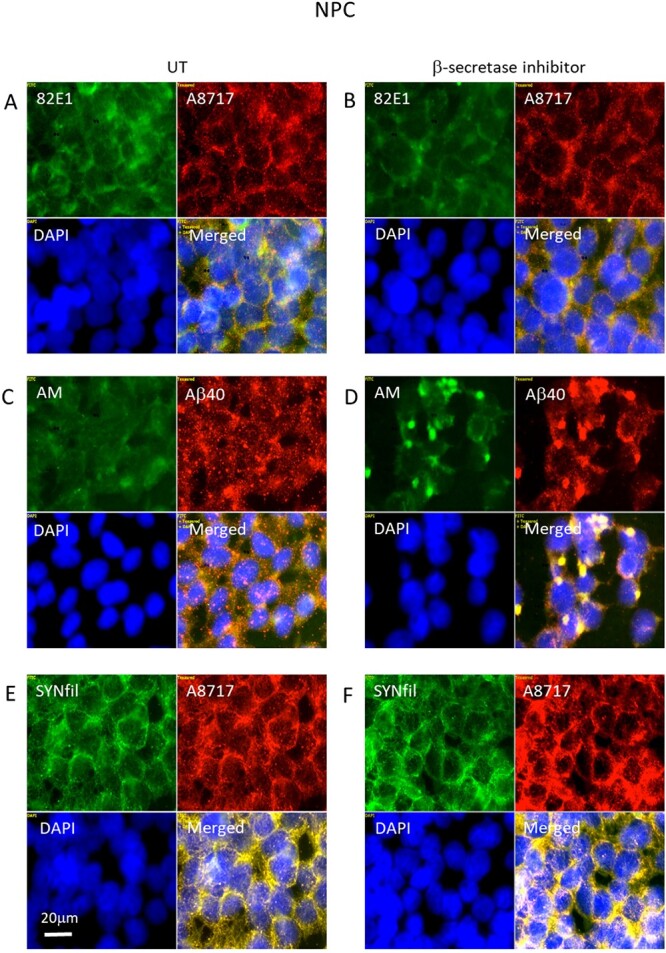
Inhibition of β-secretase suppresses formation of β-CTF and induces coclustering of HS-anMan and Αβ. Representative immunofluorescence images of cells that were grown to confluence in A, C, E) regular medium (UT, untreated) or in B, D, F) medium containing 100 nM of β-secretase inhibitor (LY2811376). Staining was performed with mAb 82E1 (for the N-terminal of β-CTF/Αβ, green), mAb AM (for HS-anMan, green), mAb SYNfil (green), pAb A8717 (for the C-terminal of APP/β-CTF, red), pAb Aβ40 (for the Aβ region, red), and DAPI (for nuclei, blue). Exposure time was the same in all cases. Bar, 20 μm.

To explore possible connections between APP and GPC1 processing and aggregation of SYN, we first inhibited APP processing by using the β-secretase inhibitor LY2811376. Cleavage of APP by β-secretase generates β-NTF and β-CTF (Fig. 1A). Cleavage was demonstrated by deconvolution immunofluorescence microscopy using mAb 82E1, which recognizes the N-terminal of β-CTF. The C-terminal of β-CTF was detected by using polyclonal antibody (pAb) A8717. Colocalization of the 82E1 and A8717 staining was taken as evidence for the presence of β-CTF. HS-anMan was detected by mAb AM and SYN filamentous (SYNfil) aggregates by using a conformation-specific rabbit mAb recognizing a SYN conformer that is known to participate in filament formation. Cells were inspected both at low and at high magnifications. As expected, growth in the presence of the β-secretase inhibitor reduced the 82E1 staining by approx. 50% (Supplementary Fig. S1A and B, 82E1/DNA-staining compound 4,6-diaminido-2-phenylindole [DAPI] 0.64 and 0.28, respectively). Staining for HS-anMan was reduced to approx. 40% (Supplementary Fig. S1C and D, AM/DAPI 0.70 and 0.38, respectively), while SYNfil staining, which was present in untreated cells, was less affected (Supplementary Fig. S1E and F, SYNfil/DAPI 0.95 and 0.78, respectively). This was confirmed by slot immunoblotting of radio-immunoprecipitationassay (RIPA) extracts of untreated and treated cells. There was a significant decrease of both 82E1 and AM immunoreactivity (Supplementary Fig. S1G, 44% of UT; Supplementary Fig. S1H, 43% of UT), while there was no significant effect on GPC1 expression (Supplementary Fig. S1I) or on SYNfil formation (Supplementary Fig. S1J). Immunofluorescence microscopy at high magnification showed reduced formation of β-CTF (Fig. 2A and B, from yellow to orange in merged). Remaining HS-anMan coclustered extensively with staining for Aβ (cf. Fig. 2C and D, merged; from diffuse orange to yellow staining that was concentrated to certain sites in the cytoplasm). The distribution of the staining for the C-terminal of APP (A8717) appeared unchanged (Fig. 2A, B, E, and F, red), indicating no clustering of β-CTF. Therefore, the clusters in Fig. 2D probably contained HS-anMan and Aβ. The distribution of SYNfil staining appeared to be unaffected and to be colocalized with staining for the C-terminal of APP/β-CTF (Fig. 2E and F, yellow in merged).

**Fig. 3:**
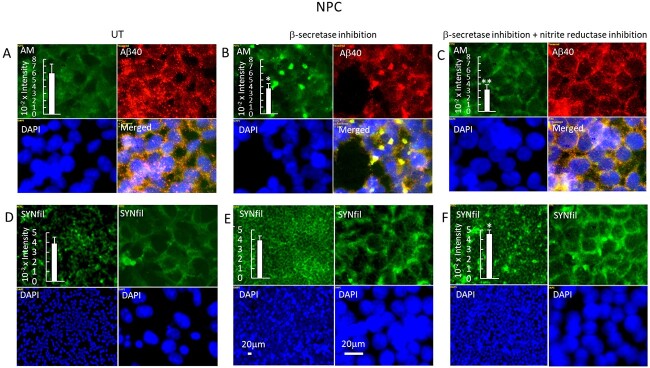
Inhibition of nitrite reduction abolishes coclustering of HS-anMan and Aβ and increases SYNfil formation during growth in the presence of the β-secretase inhibitor. Representative immunofluorescence images of cells that were grown to confluence in A, D) regular medium (UT, untreated), or in B, E) medium containing 100 nM of β-inhibitor (LY2811376), or in C, F) medium containing 100 nM β-inhibitor (LY2811376), 150 μM allopurinol, and 1 μM SB203580. Staining was performed with mAb AM (for HS-anMan, green), mAb SYNfil (green), pAb Aβ40 (for the Aβ region, red), and DAPI (for nuclei, blue). Exposure time was the same in all cases. Bar, 20 μm. Inserts, immunoblots of RIPA extracts of cells that were grown to confluence in regular medium (UT) or in medium containing the β-secretase inhibitor or in medium containing both the β-secretase inhibitor and the nitrite reductase inhibitors. The extracts were slot blotted to PVDF membranes which were probed with A–C) mAb AM, or D–F) mAb SYNfil. The error bars indicate SE.

Inhibition of APP processing also reduces the formation of β-NTF, which is necessary for the NO-dependent release of HS-anMan from GPC1 (Fig. 1A). We have previously reported that fibroblasts from the Tg2576 AD mouse model maintain a high level of HS-anMan formation by a stress-activated generation of NO from endogenous nitrite, which may counteract the effect of the β-secretase inhibitor (Cheng et al. 2016). Xanthine oxidoreductase can catalyze the reduction of nitrite to NO and the activity increases when the protein is phosphorylated, which is partly catalyzed by p38 mitogen-activated protein kinase. The oxidoreductase can be inhibited by allopurinol and the kinase by the compound SB203580 (Kayyali et al. 2001; Cantu-Medellin and Kelley 2013). To test if β-secretase inhibition had activated nitrite reduction, we exposed cells to both allopurinol and SB203580 during growth in the presence of the β-secretase inhibitor. This resulted in disappearance of the coclustering of HS-anMan and Aβ (Fig. 3B and C) and a slight increase in SYNfil formation (Fig. 3E and F).

### Modulation of APP and GPC1 processing and SYNfil formation in NPC

In these experiments, we modulated GPC1 processing in 2 ways. Deaminative release of HS-anMan from the GPC1 proteoglycan requires continuous supply of NO (Fig. 1A). To inhibit NO formation, cells were grown in the presence of the selective neural NO-synthase inhibitor S-methyl-L-thiocitrullin (SMTC) (Melikian et al. 2009). To stimulate the β-NTF-dependent release of HS-anMan, cells were grown in the presence of ascorbate (Fig. 1A). Cells were inspected by immunofluorescence microscopy, and cell lysates were assayed by slot immunoblotting.

**Fig. 4:**
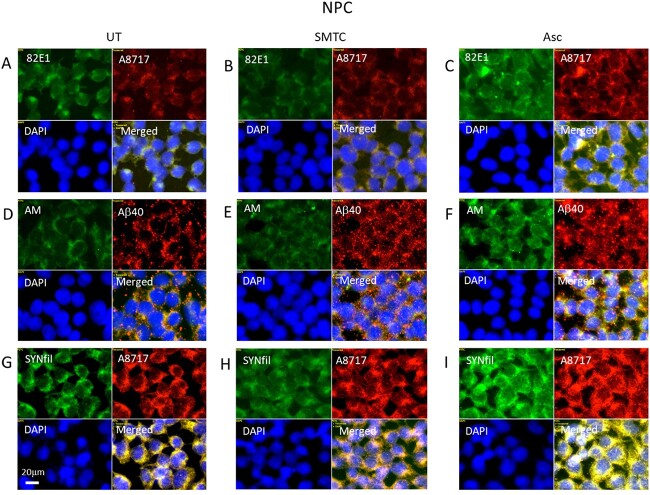
Modulation of APP and GPC1 processing and SYNfil formation. Representative immunofluorescence images of cells that were grown to confluence in A, D, G) regular medium (UT, untreated) or in B, E, H) medium containing100 μM SMTC or in C, F, I) medium containing 1 mM ascorbate (Asc). Staining was performed with mAb 82E1 (for the N-terminal of β-CTF/Αβ, green), mAb AM (for HS-anMan, green), mAb SYNfil (green), pAb A8717 (for the C-terminal of APP/β-CTF, red), pAb Aβ40 (for the Aβ region of APP/β-CTF, red), and DAPI (for nuclei, blue). Exposure time was the same in all cases. Bar, 20 μm.

NO deprivation suppressed APP processing to β-CTF (Fig. 4A and B, merged; Supplementary Fig. S2A and B, merged, and Supplementary Fig. S2J) as well as formation of HS-anMan (Fig. 4D and E; Supplementary Fig. S2D, E, and K). There was no effect on the GPC1 expression (Supplementary Fig. S2L), while SYNfil staining increased slightly (Fig. 4G and H; Supplementary Fig. S2G, H, and M).

When ascorbate was included in the medium, there was no significant stimulation of the formation of β-CTF (Fig. 4A and C; Supplementary Fig. S2A, C, and J). However, the formation of HS-anMan increased 2-fold (Fig. 4D and F; Supplementary Fig. S2D and F, and K), while GPC1 expression was unchanged (Supplementary Fig. S2L). SYNfil staining also increased (Fig. 4G and I; Supplementary Fig. S2G, I, and M) and continued to colocalize with the C-terminal of APP/β-CTF (Fig. 4G and I, merged).

### Simultaneous cytokine IL-6-stimulated APP and GPC1 processing and SYNfil formation in NPC

**Fig. 5:**
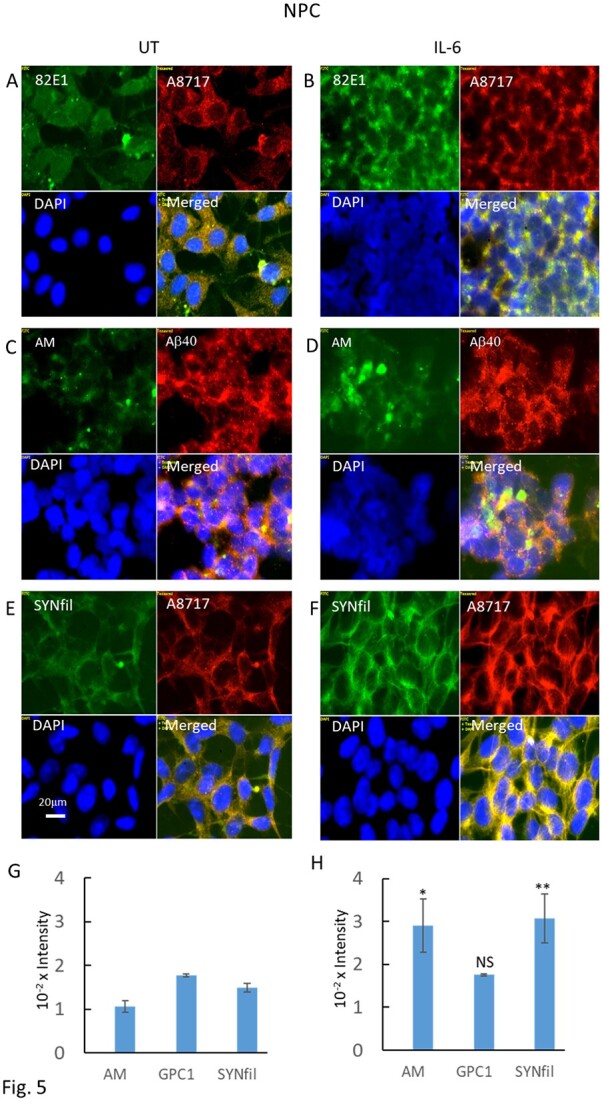
IL-6 induces increased formation of β-CTF and HS-anMan and coclustering of HS-anMan and Aβ and of β-CTF and SYNfil. Representative immunofluorescence images of cells that were grown to confluence in A, C, E) regular medium (UT, untreated) or in B, D, F) medium containing 100 ng/mL IL-6. Staining was performed with mAb 82E1 (for the N-terminal of β-CTF/Αβ, green), mAb AM (for HS-anMan, green), mAb SYNfil (green), pAb A8717 (for the C-terminal of APP/β-CTF, red), pAb Aβ40 (for the Aβ region of APP/β-CTF, red), and DAPI (for nuclei, blue). Exposure time was the same in all cases. Bar, 20 μm. G, H) Immunoblots of RIPA extracts of cells that were grown to confluence in regular medium (UT) or in medium containing 100 ng/mL of IL-6. The extracts were slot blotted to PVDF membranes which were probed with mAb AM, pAb GPC1, or mAb SYNfil. The error bars indicate SE.

Proinflammatory cytokines are known to stimulate the expression of β-secretase, which should result in increased cleavage of APP into β-NTF and β-CTF (Fig. 1A; for reviews, see e.g. Heneka et al. 2015; Calsolaro and Edison 2016; Joshi and Singh 2018; Rocha et al. 2018; see also Cheng et al. 2020 and refs. therein). Here, NPCs were grown to confluence in the absence or presence of the cytokines, TNF-α, IL-1β, or IL-6, and were assayed for APP processing by slot immunoblotting using mAb 82E1. Growth in the presence of either of the 3 cytokines resulted in increased β-secretase cleavage (Supplementary Fig. S3A). The greatest effect was obtained with IL-6 (an approx. 2.5-fold increase in 82E1 staining). Immunofluorescence microscopy of IL-6 treated cultures showed an increased clustering of β-CTF-containing cells (Fig. 5A and B; Supplementary Fig. S3B and C, yellow in merged).

As β-cleavage of APP also generates β-NTF, which can initiate deaminative release of HS from GPC1, we also stained untreated and IL-6-treated cells for HS-anMan using mAb AM and costained with both pAb Aβ40 and pAb SYN, respectively. Immunofluorescence microscopy showed clustering of HS-anMan, which partially overlapped with staining for Aβ (Fig. 5C and D and Supplementary Fig. S4A and B, green/yellow in merged), and slot immunoblotting indicated an almost 3-fold increase in HS-anMan (Fig. 5H, AM), while the expression of GPC1 was unchanged (Fig. 5H, GPC1). Moreover, HS-anMan colocalized partially with SYN (Supplementary Fig. S4D).

IL-6 treatment also resulted in an approx. 2-fold increase in SYNfil formation (Fig. 5F and H, SYNfil, and Supplementary Fig. S4F, green). The increased SYNfil staining colocalized with staining for the C-terminal of APP/β-CTF (Fig. 5F and Supplementary Fig. S4F, yellow in merged).

### Simultaneous suppression of APP and GPC1 processing and SYNfil formation in IL-6-treated NPC

**Fig. 6:**
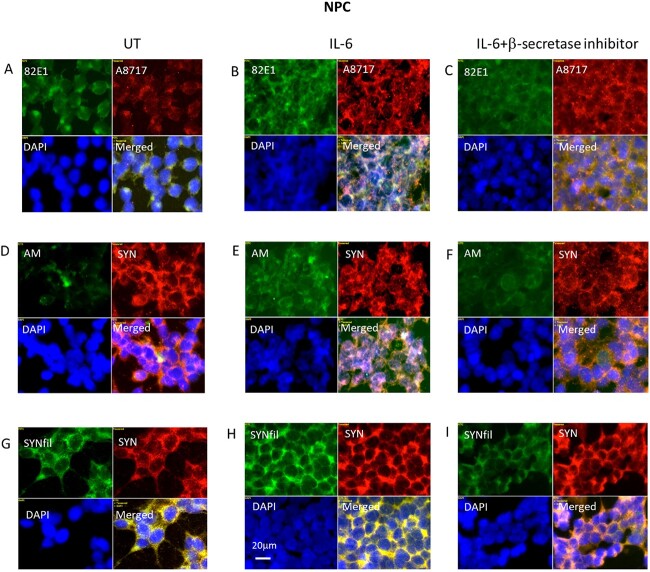
Inhibition of β-secretase suppresses IL-6-induced formation of β-CTF, HS-anMan, and SYNfil. Representative immunofluorescence images of cells that were grown to confluence in A, D, G) regular medium or in B, E, H) medium containing 100 ng/mL IL-6 or in C, F, I) medium containing 100 ng/mL IL-6 and 100 nM β-inhibitor (LY2811376). Staining was performed with mAb 82E1 (for the N-terminal of β-CTF/Αβ, green), mAb AM (for HS-anMan, green), mAb SYNfil (green), pAb A8717 (for the C-terminal of APP/β-CTF, red), pAb SYN (red), and DAPI (for nuclei, blue). Exposure time was the same in all cases. Bar, 20 μm.

To suppress cytokine-stimulated APP processing, we used again the β-secretase inhibitor LY2811376, which should diminish the formation of β-NTF and β-CTF. When cells were grown in the presence of both IL-6 and the β-secretase inhibitor, there was an approx. 30% decrease in the staining intensity of 82E1 (Supplementary Fig. S5A and B), an approx. 20% decrease in AM staining (Supplementary Fig. S5C and D), and an approx. 50% decrease in SYNfil staining (Supplementary Fig. S5E and F). High-magnification immunofluorescence images indicated that the clustering of β-CTF as well as of HS-anMan had been almost completely abolished (Fig. 6A–F, green). Reduction of HS-anMan staining should be due to diminished formation of β-NTF (Cheng et al. 2014, 2017a and 2017b). A8717 staining remained essentially unaffected, as this pAb recognizes the C-terminal of both APP and β-CTF (Fig. 6B and C, red). SYN staining also appeared to be unaffected (Fig. 6D–I, red), while staining for SYNfil was greatly reduced (Fig. 6H and I, red). Taken together, an increased APP processing is accompanied by an increased SYNfil formation, and decreased APP processing is accompanied by decreased SYNfil formation.

### Modulation of HS-anMan formation prevents increased SYNfil formation in IL-6-treated NPC

**Fig. 7:**
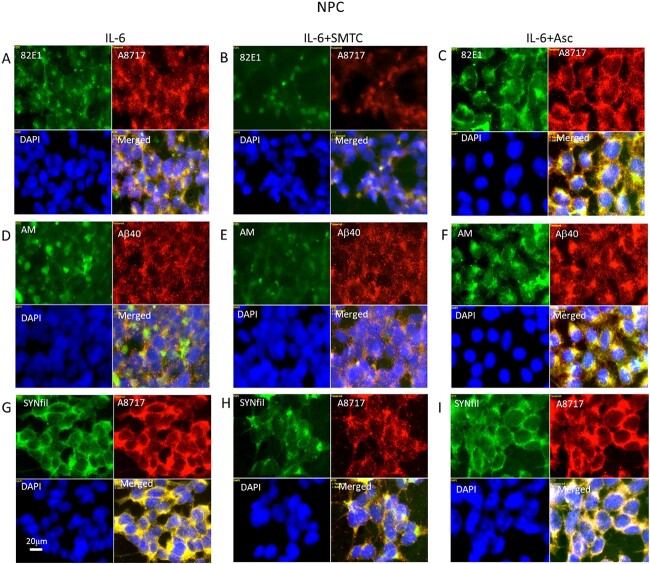
NO deprivation suppresses and ascorbate stimulates APP processing and HS-anMan formation in IL-6-treated cells. Representative immunofluorescence images of cells that were grown to confluence in A, D, G) medium containing 100 ng/mL IL-6 or in B, E, H) medium containing 100 ng/mL IL-6 and 100 μM SMTC or in C, F, I) medium containing 100 ng/mL IL-6 and 1 mM ascorbate (Asc). Staining was performed with mAb 82E1 (for the N-terminal of β-CTF/Αβ, green), mAb AM (for HS-anMan, green), mAb SYNfil (green), pAb A8717 (for the C-terminal of APP/β-CTF, red), pAb Aβ40 (for the Αβ region, red), and DAPI (for nuclei, blue). Exposure time was the same in all cases. Bar, 20 μm.

To evaluate the contribution of HS-anMan, we separately suppressed or increased its formation. Deaminative release of HS-anMan from the GPC1 proteoglycan requires continuous supply of NO (Fig. 1). To inhibit NO formation in IL-6-treated cells, they were grown in the presence of IL-6 and the selective neural NO-synthase inhibitor SMTC (Melikian et al. 2009). Low-magnification images indicated an approx. 40% reduction of 82E1 staining intensity (Supplementary Fig. S6A and B), an approx. 50% suppression of HS-anMan formation (Supplementary Fig. S6D and E), and an approx. 20% reduction of SYNfil staining intensity (Supplementary Fig. S6G and H). High-magnification images showed a reduced clustering of β-CTF (Fig. 7A and B, green and merged), a reduced coclustering of HS-anMan and Aβ (Fig. 7D and E, green and merged), and a reduced colocalization of SYNfil with the staining for the C-terminal of APP/β-CTF (Fig. 7G and H, green and merged). However, the decrease in β-CTF and SYNfil colocalization was less pronounced as compared to β-inhibition (cf. Figure 6I).

Ascorbate stimulates the β-NTF-dependent deaminative release of HS-anMan from GPC1 (Fig. 1A). When NPCs were grown in the presence of IL-6 and ascorbate, low-magnification images indicated a 2-fold increase in 82E1 staining intensity and a 2.5-fold increase in HS-anMan formation (Supplementary Fig. S6C and F, respectively). However, there was no significant change in SYNfil staining (Supplementary Fig. S6I). High-magnification images showed more uniform presence of β-CTF (Fig. 7C, merged) and more uniform colocalization of HS-anMan and Aβ (Fig. 7F, merged).

### Ascorbate is critical for differentiation of NPC to induced differentiated NPC

**Fig. 8:**
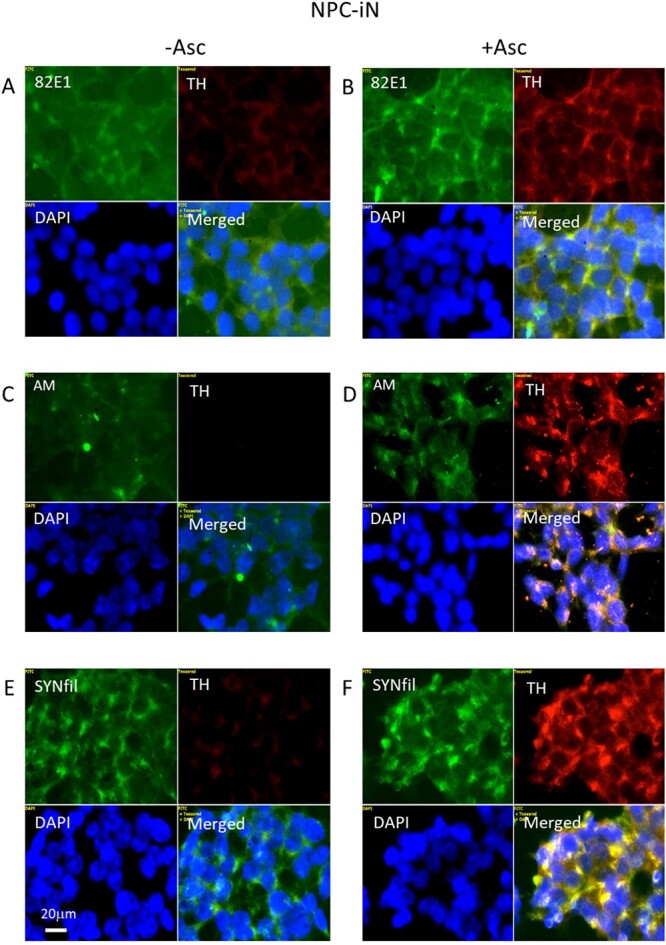
Differentiation of NPC to NPC-iN requires the presence of ascorbate in the medium. Representative immunofluorescence images of cells that were maintained in A, C, E) differentiation medium without added ascorbate (-Asc) or with B, D, F) 0.1 μM ascorbate (+Asc). Staining was performed with mAb 82E1 (for the N-terminal of β-CTF/Αβ, green), mAb AM (for HS-anMan, green), mAb SYNfil (green), pAb anti-TH (red), and DAPI (for nuclei, blue). Exposure time was the same in all cases. Bar, 20 μm.

Differentiation of NPC to induced neurons (induced differentiated NPC [NPC-iN]) can be obtained by maintaining a confluent cell culture in a special medium that contains a small amount of ascorbate (0.1 μM). Expression of TH, which is a prerequisite for the formation of dopamine, was taken as evidence for a successful differentiation. When ascorbate was omitted from the differentiation medium, staining for TH was very weak or almost undetectable (Fig. 8A, C, and E; frame TH; see also Supplementary Fig. S7A and C). However, when ascorbate was included in the medium, there was an expression of TH (Fig. 8B, D, and F; TH, red; see also Supplementary Fig. S7B and D). There was also a sporadic overlap between 82E1 and TH staining (Supplementary Fig. S7B and Fig. 8B, both green and yellow in merged), while SYNfil and TH showed a more distinct colocalization (Supplementary Fig. S7D and Fig. 8F, yellow in merged). Colocalization between HS-anMan and TH was less pronounced (Fig. 8D, merged).

### Suppression of HS-anMan release from GPC1 reduces TH staining and induces coclustering of HS-anMan and SYNfil with TH in differentiating NPC (NPC-iN)

**Fig. 9:**
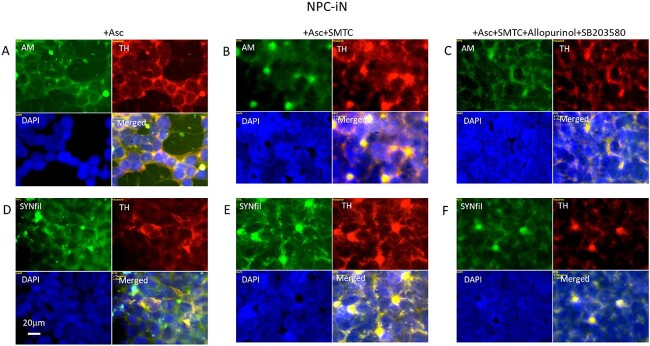
Suppression of HS-anMan release from GPC1 during differentiation of NPC to NPC-iN induces coclustering of SYNfil and TH. Representative immunofluorescence images of cells that were maintained in A, D) complete (+0.1 μM Asc) differentiation medium without further additions, or in B, E) complete medium containing 100 μM SMTC, or in C, F) complete medium containing 100 μM SMTC, 150 μM allopurinol, and 1 μM SB203580. Staining was performed with mAb AM (for HS-anMan, green), mAb SYNfil (green), pAb anti-TH (red), and DAPI (for nuclei, blue). Exposure time was the same in all cases. Bar, 20 μm.

To suppress the NO-dependent deaminative release of HS-anMan from GPC1, we inhibited NO formation by using SMTC (NO-synthase inhibitor) and allopurinol + SB203580 (nitrite reductase-inhibition). A combination of both treatments significantly reduced TH staining intensity (Supplementary Fig. S8). Immunofluorescence microscopy revealed coclustering of HS-anMan with TH and of SYNfil with TH after inhibition of NO-synthase (Fig. 9B and E, yellow in merged). After additional inhibition of nitrite reductase, HS-anMan clustering decreased (Fig. 9C, green) and SYNfil coclustering with TH was also less pronounced (Fig. 9F, merged).

### Suppression of APP processing induces clustering of APP degradation products, TH, HS-anMan, and SYNfil in differentiating NPC (NPC-iN)

**Fig. 10:**
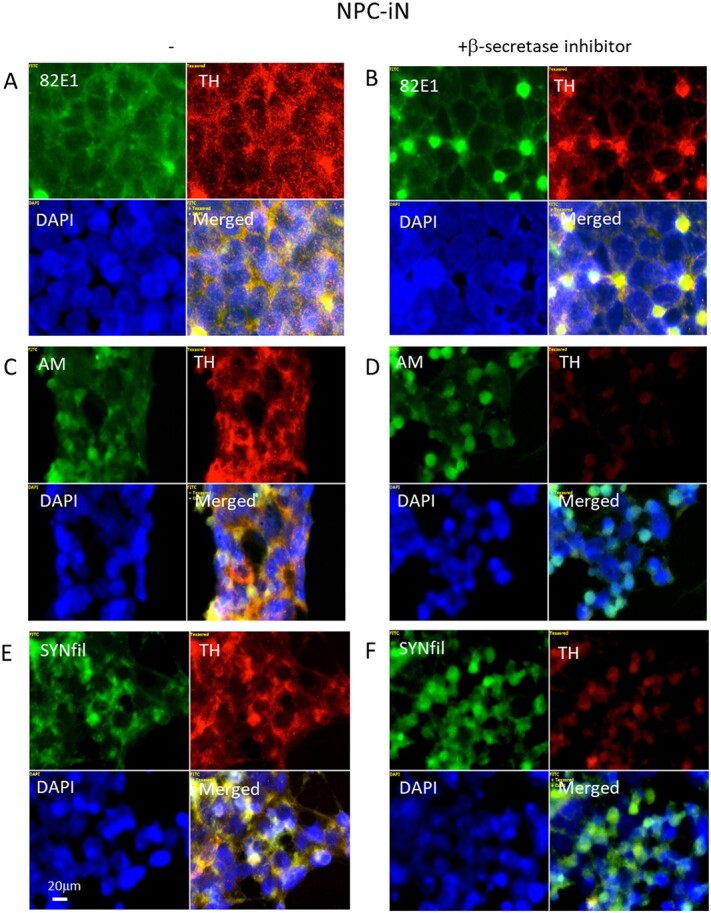
Suppression of APP processing induces clustering of APP degradation products, TH, HS-anMan, and SYNfil in differentiating cells (NPC-iN). Representative immunofluorescence images of cells that were maintained in A, C, E) complete (+0.1 μM Asc) differentiation medium without further additions (−) or in B, D, F) complete medium containing 100 nM LY2811376 (+β-inhibitor). Staining was performed with mAb 82E1 (for the N-terminal of β-CTF/Αβ, green), mAb AM (for HS-anMan, green), mAb SYNfil (green), pAb anti-TH (red), and DAPI (for nuclei, blue). Exposure time was the same in all cases. Bar, 20 μm.

To suppress APP processing, the β-secretase inhibitor LY2811376 was included in the complete differentiation medium, and cells were examined by immunofluorescence microscopy. When APP processing was suppressed in NPC-iN, the 82E1-positive APP degradation products, HS-anMan (AM), and SYNfil all clustered extensively (Fig. 10B, D, and F, respectively, green). The APP degradations products also colocalized with TH staining (Fig. 10B, yellow in merged). The TH, AM, and SYNfil staining partly overlapped with DAPI, which may indicate that inhibition of APP processing is detrimental to the viability of NPC-iN.

### The cytokine IL-6 induced coclustering of APP degradation products, HS-anMan, and SYNfil with TH can be reversed by excess ascorbate in differentiating NPC (NPC-iN)

**Fig. 11:**
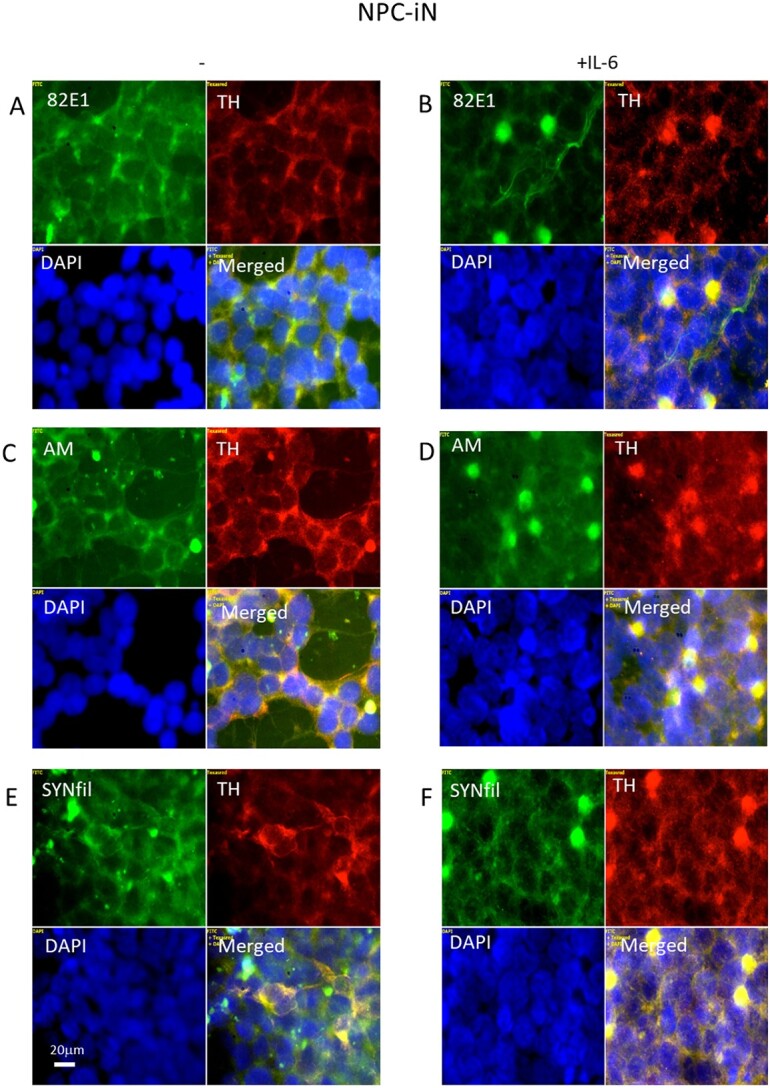
IL-6 induces clustering of APP degradation products, HS-anMan, SYNfil, and TH in differentiating cells (NPC-iN). Representative immunofluorescence images of cells that were maintained in A, C, E) complete (+0.1 μM Asc) differentiation medium without further additions (−) or in B, D. F) complete medium containing 100 ng/mL IL-6. Staining was performed with mAb 82E1 (for the N-terminal of β-CTF/Αβ, green), mAb AM (for HS-anMan, green), mAb SYNfil (green), pAb anti-TH (red), and DAPI (for nuclei, blue). Exposure time was the same in all cases. Bar, 20 μm.

**Fig. 12:**
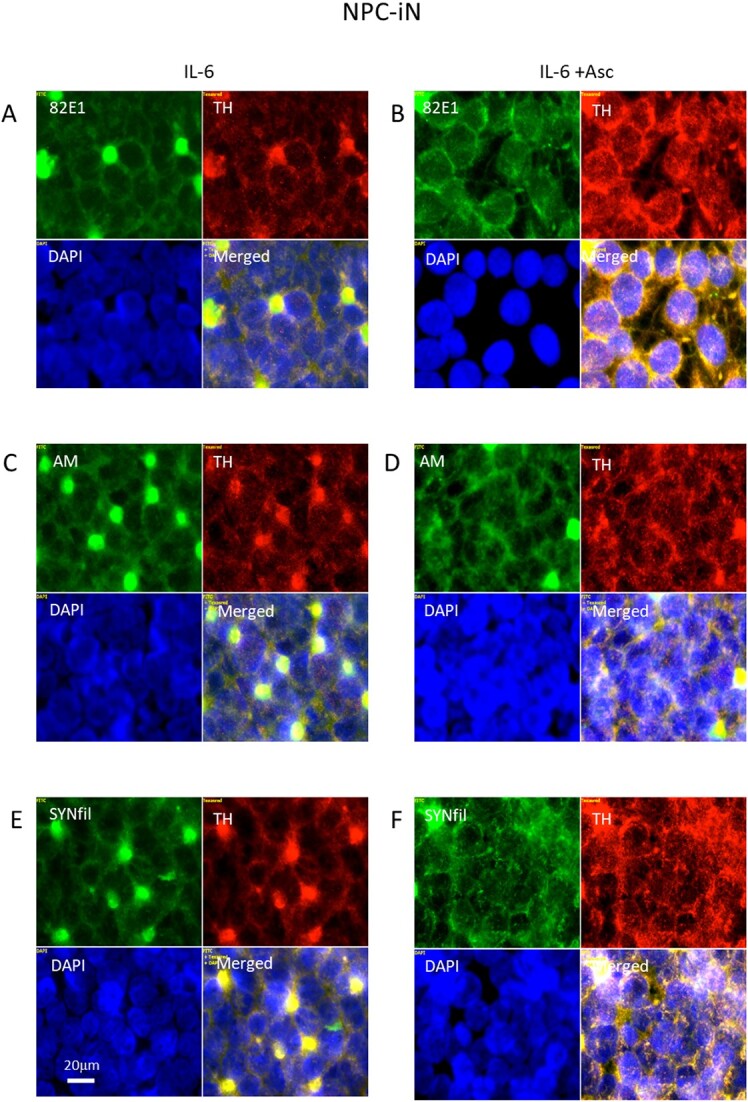
Reversal of IL-6-induced clustering of APP degradation products, HS-anMan, SYNfil, and TH by excess ascorbate. Representative immunofluorescence images of cells that were maintained in A, C, E) complete (+0.1 μM Asc) differentiation medium containing 100 ng/mL IL-6 (IL-6) or in B, D, F) complete (+0.1 μM Asc) differentiation medium containing100 ng/mL IL-6 and 1 mM ascorbate (IL-6 + Asc). Staining was performed with mAb 82E1 (for the N-terminal of β-CTF/Αβ, green), mAb AM (for HS-anMan, green), mAb SYNfil (green), pAb anti-TH (red), and DAPI (for nuclei, blue). Exposure time was the same in all cases. Bar, 20 μm.

To increase APP processing during differentiation, IL-6 was included in the complete differentiation medium. This resulted in clustering of APP degradation products (Fig. 11B, 82E1, green), of HS-anMan released from GPC1 (Fig. 11D, AM, green), and of SYNfil (Fig. 11F, green), all of which coclustered with TH (Fig. 11B, D, and F, merged). To stimulate HS-anMan formation, an excess of ascorbate (1 mM) was included in the IL-6-containing differentiation medium. This resulted in reduced clustering (Fig. 12B, D, and F, merged).

### IL-6-induced clusters of SYNfil colocalize with autophagosome marker in differentiating NPC (NPC-iN)

**Fig. 13:**
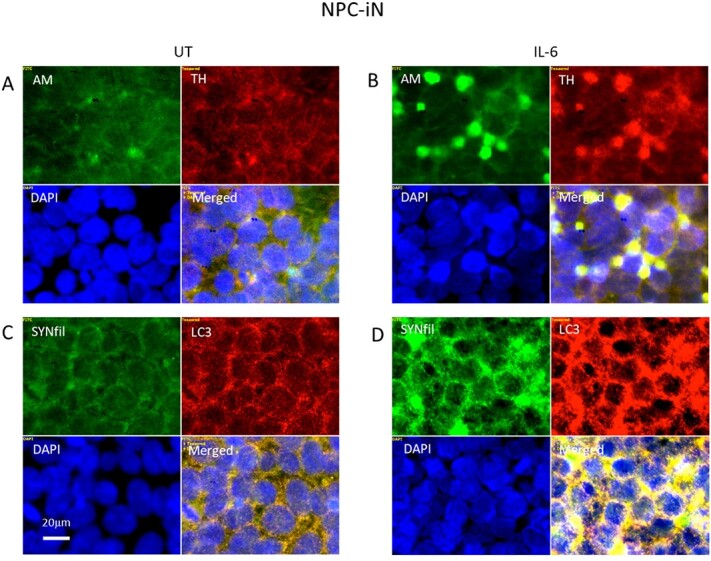
IL-6 induces colocalization of SYNfil with the autophagosome marker LC3. Representative immunofluorescence images of cells that were maintained in A, C) complete differentiation medium without further additions (−) or in B, D) complete (+ 0.1 μM Asc) medium containing 100 ng/mL IL-6. Staining was performed with mAb AM (for HS-anMan, green), mAb SYNfil (green), pAb anti-TH (red), pAb LC3 (autophagosome marker), and DAPI (for nuclei, blue). Exposure time was the same in all cases. Bar, 20 μm.

The IL-6-induced coclustering of APP degradation products, HS-anMan, SYNfil, and TH during differentiation of NPC to NPC-iN may indicate an association with autophagosomes as was previously shown for undifferentiated NPC grown in the presence of IL-6 (Cheng et al. 2022). We, therefore, performed an additional experiment where we costained with the autophagosome marker LC3. In the control, staining for HS-anMan and TH displayed again coclustering after differentiation in the presence of IL-6 (Fig. 13B, yellow in merged). IL-6 also induced SYNfil coclustering with LC3 (Fig. 13D, yellow in merged). As APP degradation products, HS-anMan, and SYNfil coclustered with TH (Fig. 11B, D, and F, yellow in merged), both SYNfil and TH may accumulate in autophagosomes together with APP degradation products and HS-anMan.

## Discussion

We have explored the possible interplay between APP, GPC1, and SYN in undifferentiated and differentiated NPC by up- and downregulation of HS-anMan formation. Control experiments showed that GPC1 was the major, if not exclusive, source of HS-anMan. Moreover, expression of GPC1 was not affected by inhibition of β-secretase or NO-synthase, or by ascorbate or by exposure to IL-6.

In undifferentiated NPC, both suppression and stimulation of HS-anMan release had a small effect on SYNfil formation. HS has a dual effect on APP processing. β-Secretase activity is inhibited by HS (Scholefield et al. 2003), thereby constituting a potential, negative feed-back loop. However, ascorbate-induced HS-anMan formation also results in endosomal acidification, which stimulates β-secretase activity, i.e. a feed-forward effect (Cheng et al. 2021). Thus, the extent of β-CTF/Aβ formation may have been insufficient to generate a major increase in SYNfil aggregation (Köppen et al. 2020).

When NPCs were grown in the presence of IL-6, there was an increased formation of β-CTF, increased release of HS-anMan from GPC1, and increased SYNfil formation. HS-anMan coclustered with Aβ, and SYNfil colocalized with the C-terminal of β-CTF. The latter and/or Aβ may thus promote SYN aggregation. Accordingly, when β-cleavage of APP was suppressed in IL-6-treated cells, SYNfil formation was also suppressed.

As mentioned above, HS-anMan may modulate SYNfil formation in 2 different ways. When release of HS-anMan from GPC1 was reduced by NO deprivation of IL-6-treated NPC, SYNfil formation was suppressed. This may be due to lowered endosomal acidification, which would decrease β-secretase activity and would result in diminished formation of β-CTF and Aβ. In a previous study, HS-deficient GPC1 was generated in IL-6 treated NPC by using a cyanobacterial neurotoxin, whereby the capacity to release HS-anMan was diminished. This resulted in decreased accumulation of SYNfil aggregates in autophagosomes and massive SYN secretion (Cheng et al. 2022).

In the present study, when NPCs were grown in the presence of IL-6 and ascorbate, release of HS-anMan from GPC1 and β-processing of APP were both greatly stimulated. This would be expected to increase SYNfil formation (Köppen et al. 2020). Instead, there was no significant effect on SYNfil staining. We have previously demonstrated the formation of stable complexes between HS-anMan and Aβ in AD mouse fibroblasts (Cheng et al. 2011) and between HS-anMan and β-CTF in TNF-α-stimulated N2a neuroblastoma cells (Cheng et al. 2020). In the present study, there was extensive overlap between HS-anMan and Aβ staining in IL-6- and ascorbate-treated NPC, which may indicate complex formation. In HS-Aβ complexes, the ability of Αβ to induce SYNfil formation may be inhibited. Accordingly, when HS-anMan formation was extensively suppressed and HS-anMan-Aβ overlap was less prevalent, SYNfil increased.

NPCs were differentiated into TH-expressing cells by maintaining a confluent culture in a special medium that contained a small amount of ascorbate as an obligatory component. Ascorbate is a cofactor for various hydroxylation reactions catalyzed by dioxygenases. These can generate various epigenetic changes in DNA that are necessary for the differentiation of NPC (He et al. 2015). Ascorbate is also, indirectly, a stimulator of APP-dependent GPC1 processing, which results in the formation of β-CTF/Aβ, potential inducers of SYNfil formation Köppen et al. 2020). The clustered SYNfil staining colocalized with TH. Moreover, suppression of HS-anMan release from GPC1 resulted in decreased staining for TH and coclustering of TH with both HS-anMan and SYNfil, suggesting a role for GPC1 in the differentiation process.

Expression of β-secretase, more specifically, β-site APP cleaving enzyme 1 is required for the differentiation of NPC to neuronal lineage (Chatila et al. 2018). In this study, inhibition of β-secretase caused extensive cell damage and clustering of APP degradation products, HS-anMan, TH, and SYNfil in keeping with a disturbed differentiation process. When IL-6 was included in the differentiation medium, there was also coclustering of APP degradation products, HS-anMan, SYNfil, and TH. As the clusters were also associated with an autophagosome marker, IL-6 may have induced the autophagy of TH, which may also disturb the differentiation process. Stimulation of GPC1 processing by including 1 mM of ascorbate reversed the IL-6-induced clustering. HS-anMan, which is ultimately captured by autophagosomes and degraded in lysosomes, may be involved in the catabolism of SYNfil and TH (Cheng et al. 2014).

Proinflammatory cytokines promote neurodegeneration in both AD and PD (for reviews, see Heneka et al. 2015; Calsolaro and Edison 2016; Joshi and Singh 2018; Rocha et al. 2018). A mixed AD-PD pathology may thus be initiated by the increased expression of β-secretase, which results in increased processing of APP into β-NTF and β-CTF. The latter is then a potential stimulator of SYN aggregation (Köppen et al. 2020). Release of HS-anMan from GPC1 is dependent on the generation of β-NTF. HS-anMan is a potential moderator of APP-dependent SYN aggregation and a possible vehicle for the transport of SYN to autophagosomes.

There are opposing views on the role of HS in neurodegenerative diseases. While full-length HS can change protein conformations into amyloidogenic forms, small HS oligosaccharides do not. We have previously shown that full-length HS chains can promote Aβ oligomerization, while HS-anMan oligosaccharides modulate or suppress oligomerization (Cheng et al. 2011, 2013, and refs. therein).

## Materials and methods

### Cells and reagents

iPSC-derived human neuronal progenitor cells (NPC, ATCC ACS-5003) were plated in CellMatrix gel-coated plates (Growth kit ACS-3003 for NPC expansion). The cells were cultured in a 1:1 mixture of Dulbecco Minimum Essential Medium and F12 Medium (ATCC 30-2006) supplemented with 10% (v/v) fetal bovine serum. Differentiation of NPC was carried out using a Dopaminergic Neuron Differentiation kit (ACS-3004), which was replaced with regular intervals according to the instructions provided by ATCC. The cytokines used were: TNF-α from Alomone labs, IL-1β from Gibco, and IL-6 from Tataabiocenter. The APP antibodies were: mAb 82E1 from IBL, pAb A8717 from Sigma and pAb Αβ40 from Thermo Fisher (PA3-16760). The SYN antibodies were: pAb SYN from Chemicon (ab5038) and rabbit mAb SYNfil from Abcam (MJFR-14-6-4-2, ab209538). mAb AM is specific for HS/heparin tetrasaccharide or larger fragments that are generated by partial deaminative cleavage and therefore terminate with anhydromannose (anMan) at the reducing end (Pejler et al. 1988). It has been characterized in previous studies (Ding et al. 2002; Mani et al. 2004) In GPC1-HS, the in vivo cleavage occurs at N-unsubstituted glucosamines (Cheng et al. 2012). A rabbit antiserum raised against human GPC1 was the same as described and used earlier ( Cheng et al. 2013). A pAb against TH was obtained from Millipore (AB152) and anti-LC3 and FITC-tagged goat anti-mouse Ig were obtained from Sigma-Aldrich. Alexa-Fluor 594-tagged donkey anti-rabbit IgG was from Invitrogen, Alexa Fluor 594-tagged goat anti-mouse IgG and OregonGreen 488-tagged goat anti-rabbit IgG were obtained from Molecular Probes, United States. The commercial antibodies were used as recommended by the manufacturers. The DAPI, allopurinol, and ascorbate were obtained from Sigma-Aldrich and LY2811376 and SB203580 were obtained from SelleckChem. SMTC was purchased from Santa Cruz.

### CRISPR/Cas9 targeting of Gpc1

Approximately, 1 × 10^5^ NPC cells were plated at each well of Cell Matrix gel-coated 4-well slides. At the level of 40%–80% confluence, the cells were transfected either with 4 μg of green fluorescent protein-tagged human *GPC1*-targeted CRISPR/Cas9 knockout plasmid (Gpc1 Double Nickase Plasmid (h): sc-402002-NIC; Santa Cruz Biotechnology, Dallas, TX, United States) or 4 μg of a similarly tagged nonspecific CRISPR/Cas9 control plasmid (not targeting any known gene; Control CRISPR/Cas9 Plasmid, sc-418922; Santa Cruz Biotechnology) according to the manufacturer’s instructions. For transfection, 10 μL of plasmid DNA:UltraCruz Transfection Reagent (sc-395739) was incubated with 1 μg of plasmid DNA in 50 μL of Plasmid Transfection Medium (sc-108062) for 5 min at room temperature. Prior to transfection, the culture media was replaced with 700 μL of fresh antibiotic-free Plasmid Transfection Medium. Then, 50 μL of Plasmid DNA/UltraCruz Transfection Reagent Complex was added dropwise to each well and the cells were incubated for 24–72 h posttransfection.

### Deconvolution immunofluorescence microscopy

Cells were examined by immunofluorescence microscopy as described previously (Cheng et al. 2014). In brief, cells were fixed in acetone in order to retain cellular and subcellular structures and to ensure the preservation of carbohydrates. The fixed cells were first precoated with 10% anti-mouse total Ig and were then exposed to primary antibodies overnight. The secondary antibodies used were FITC-tagged goat antimouse Ig or OregonGreen 488-tagged goat anti-rabbit IgG, when the primary antibody was a monoclonal and Alexa Fluor 594-tagged goat anti-rabbit IgG, or sometimes, Alexa Fluor 594-tagged donkey anti-goat IgG when the primary antibody was a polyclonal. In the controls, the primary antibody was omitted. DNA staining with DAPI, as well as staining with antibodies, was performed as recommended by the manufacturers. The fluorescent images were analyzed by using a Carl Zeiss AxioObserver inverted fluorescence microscope with deconvolution technique and equipped with objective EC “Plan-Neofluar” 63×/1.25 Oil M27 and AxioCam MRm Rev Camera. Identical exposure settings and times were used for all images. During microscopy, the entire slides were scanned and immunofluorescence images at 20× and 100× magnifications were captured. The low-magnification images were used to identify representative locations for high-magnification images and for intensity measurements. Immunofluorescence signal in 5 randomly chosen low-magnification areas were quantified by densitometry using Zeiss ZEN 3.5 pro blue edition software. The results were presented in graphs representative for each experiment, *n* = 5 in each experiment. The data points are shown as the means ± SD or ±SE as indicated in the figure legends.

### Slot blot

Cells (10^6^ cells) were extracted with radio-immunoprecipitation assay (RIPA) buffer (0.1% w/v SDS, 0.5% v/v Triton X-100, and 0.5% w/v sodium deoxycholate in PBS supplemented with 0.5 mM phenylmethylsulfonyl fluoride at 4 °C. Protein concentration in the RIPA extracts were measured using Nanodrop (NanoVue plus). RIPA extracts containing equal amount of protein were transferred to PVDF membranes using slot blot. The PVDF membranes were then incubated with different antibodies followed by visualization using horseradish peroxidase-conjugated goat anti-mouse IgG (Bio-Rad, Hercules, CA, United States; 172-1011; dilution 1:1,000) for mAbs or horseradish peroxidase-conjugated goat anti-rabbit IgG (Bio-Rad, Hercules, CA, United States; 170-6515; dilution 1:1,000) for pAbs. The membranes were then washed extensively with PBS containing 0.5% Tween-20. The membranes were further developed by chemiluminescence (Pierce fast western blot kit) using Amersham ImageQuant 500 detector from Cytiva. Staining intensities were recorded by densitometry using GelAnalyzer 19.1 (www.gelanalyzer.com). The results were presented in graphs representative for each experiment, *n* = 5 in each experiment. The data points are shown as the means ± SD.

### Statistical methods

The data points in the graphs are shown as the means ± SD or ±SE (error bars) as indicated in the figure legends, *n* = 5 in each experiment. For statistical analysis, 2 group comparisons were performed using unpaired 2-tailed student *t*-test and unequal variances data analysis. Error probabilities of *P* < 0.05 were considered to be statistically significant. Indication of *P*-value summaries: N.S. (not significant) *P* > 0.05, ^*^P ≤ 0.05, ^*^^*^P ≤ 0.01, ^*^^*^^*^P ≤ 0.001, and ^*^^*^^*^^*^P ≤ 0.0001.

## Abbreviations

Aβ, amyloid beta peptides; anMan/AM, anhydromannose; APP, amyloid precursor protein; AD, Alzheimer’s disease; CTF, C-terminal fragment; DAPI, DNA-staining compound 4,6-diaminido-2-phenylindole; GPC1, glypican-1; HS, heparan sulfate; IL-1β, interleukin-1-beta; IL-6, interleukin-6; mAb, monoclonal antibody; NO, nitric oxide; NPC, neural progenitor cell; NPC-iN, induced differentiated NPC; NTF, N-terminal fragment; pAb, polyclonal antibody; PD, Parkinson’s disease; RIPA, radio-immunoprecipitation assay; SMTC, S-methyl-L-thiocitrullin; SNO, S-nitrosothiol; SYN, α-synuclein; SYNfil, filamentous SYN; TH, tyrosine hydroxylase; TNF-α, tumor necrosis factor-alpha.

## Supplementary Material

Supplementary_material-GLYCO-2022-00098_R1_cwad013

## Data Availability

The datasets generated during and/or analyzed during the current study are available from the corresponding author upon reasonable request.
