# Supplementary material for: Interplay between APP and glypican-1 processing and α-synuclein aggregation in undifferentiated and differentiated human neural progenitor cells
Source: Glycobiology. 2023 Feb 15;33(4):325–41. doi: 10.1093/glycob/cwad013 (PMC10208675; doi:10.1093/glycob/cwad013)
Supplement: Supplementary_material-GLYCO-2022-00098_R1_cwad013 [file supplementary_material-glyco-2022-00098_r1_cwad013.pdf]

Supplementary material

Interplay between APP and glypican-1 (GPC1) processing and α-synuclein (SYN) aggregation in undifferentiated and differentiated human neural progenitor cells (NPC)

Fang Cheng, Lars-Åke Fransson and Katrin Mani*

Department of Experimental Medical Science, Division of Neuroscience, Glycobiology Group, Lund University, Biomedical Center A13, SE-221 84 Lund, Sweden

∗To whom correspondence should be addressed: Tel: +46-46-222-4044; e-mail: katrin.mani@med.lu.se

Key words: Amyloid precursor protein/Glypican-1/Heparan sulfate/ Parkinson´s disease/ α-Synuclein

Running head: Amyloid precursor protein, glypican-1 and α-synuclein interplay

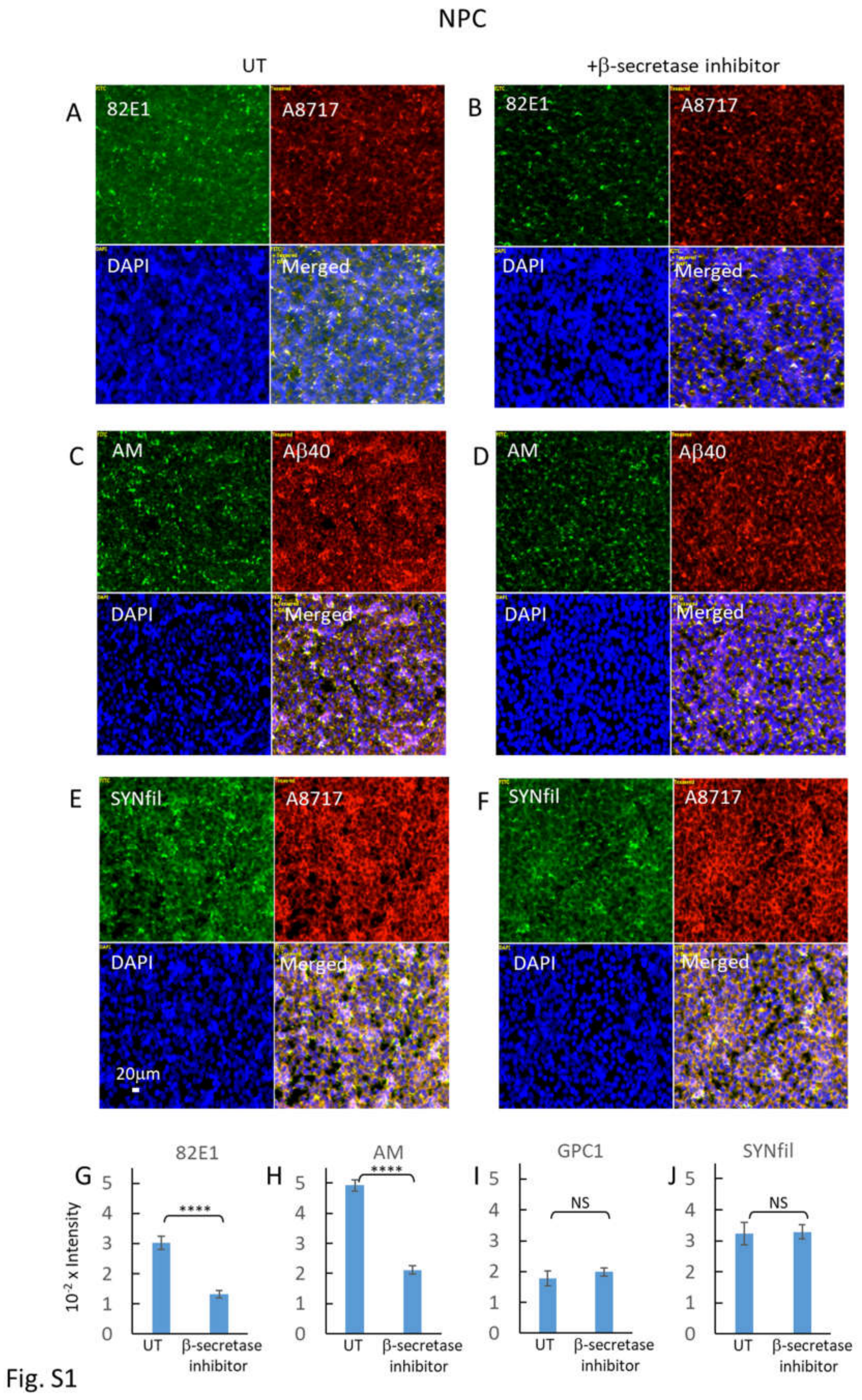

Fig. S1. Inhibition of β-secretase suppresses formation of β−CTF and HS-anMan. Low magnification immunofluorescence images of cells that were grown to confluence in regular medium (A, C, E; UT = untreated) or in medium containing 100 nM β-secretase inhibitor

(LY2811376) (B, D, F; +β-secretase inhibitior). Staining was performed with mAb 82E1 (for the N-terminal of β-CTF/Aβ, green), mAb AM (for HS-anMan, green), mAb SYNfil (green), pAb A8717 (for the C-terminal of APP/β-CTF, red), pAb Aβ40 (for the Aβ region, red), and DAPI (for nuclei, blue). The intensities of 82E1, HS-anMan, SYNfil, and DAPI staining were measured and expressed as the ratio of the respective green intensities divided by the intensity of DAPI (blue). Exposure time was the same in all cases. Bar, 20 μm. (G-J) Immunoblots of RIPA extracts of cells that were grown to confluence in regular medium (UT) or in medium containing the β-secretase inhibitor. The extracts were slot blotted to PVDF membranes which were probed with mAb 82E1 (G), mAb AM (H), pAb GPC1 (I), or mAb SYNfil (J). The error bars show the means ±SD.

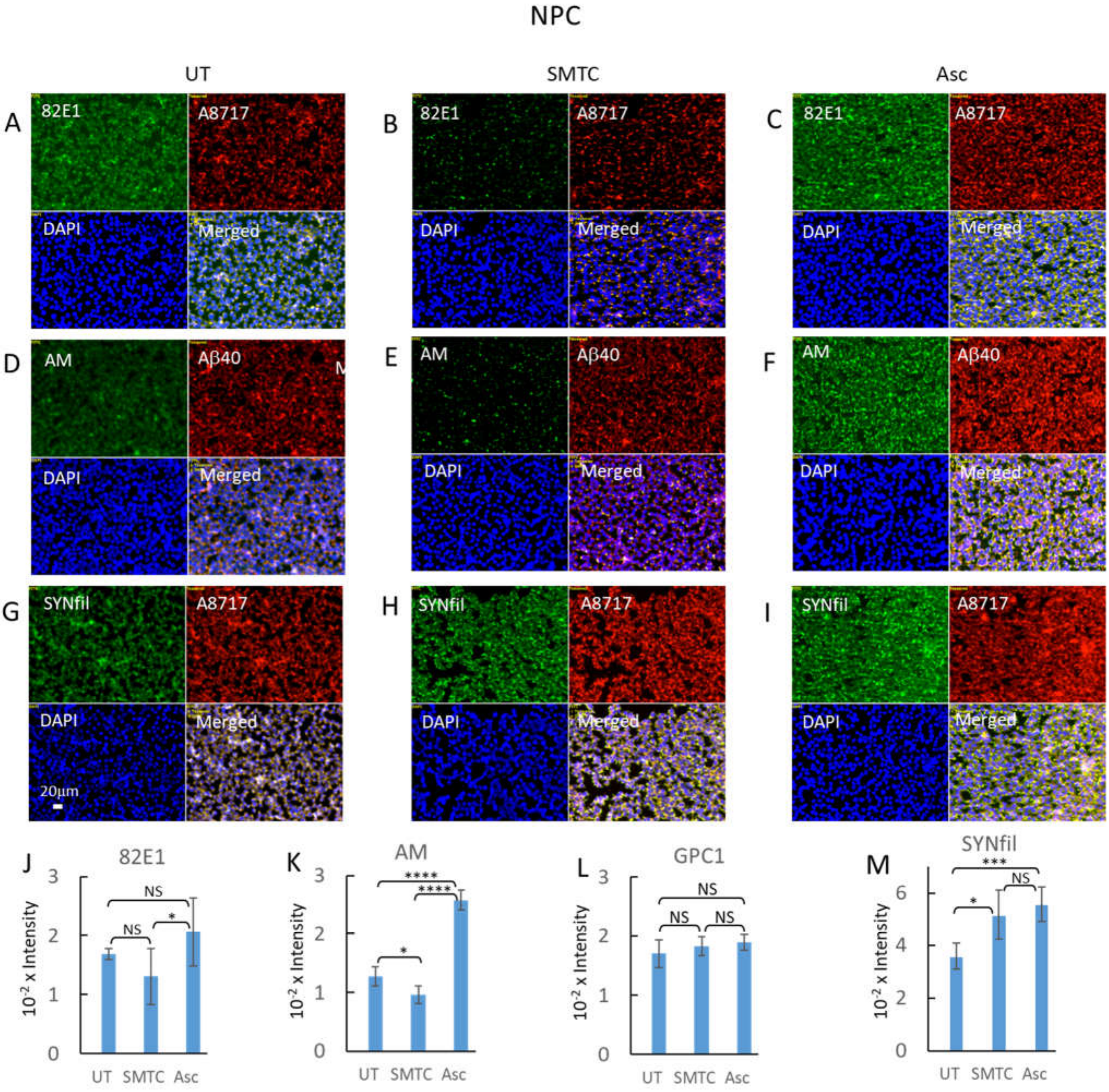

Fig. S2

Fig. S2. Modulation of APP and GPC1 processing and SYNfil formation. (A-I) Low magnification immunofluorescence images of cells that were grown to confluence in regular medium (A, D, G, UT = untreated) or in medium containing 100 µM S-methyl-L-thiocitrulline (SMTC) (B, E, H) or in medium containing 1 mM ascorbate (Asc) (C, F, I). Staining was performed with mAb 82E1 (for the N-terminal of β-CTF/Aβ, green), mAb AM (for HS-anMan, green), mAb SYNfil (green), pAb A8717 (for the C-terminal of APP/β-CTF, red), pAb Aβ40 (for the Aβ region of APP/β-CTF, red), and DAPI (for nuclei, blue). Exposure time was the same in all cases. Bar, 20 µm. (J-M) Immunoblots of RIPA extracts of cells that were grown to confluence in regular medium (UT = untreated) or in medium containing 100 µM S-methyl-L-thiocitrulline (SMTC) or in medium

containing 1 mM ascorbate (Asc). The extracts were slot blotted to PVDF membranes which were probed with mAb 82E1 (J), mAb AM (K), pAb GPC1 (L), or mAb SYNfil (M). The error bars show the means ±SD.

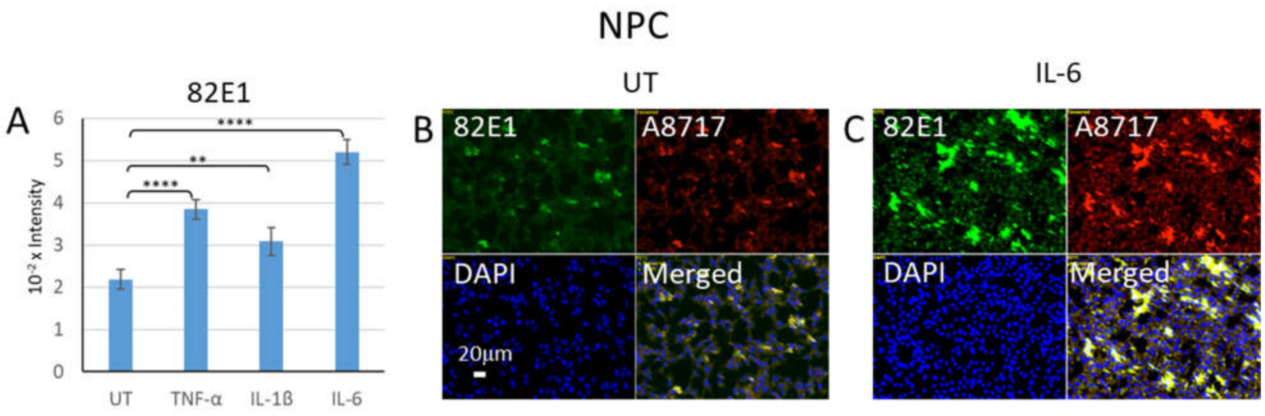

Fig. S3

Fig. S3. The cytokines TNF-α, IL-1β and IL-6 induce increased formation of β-CTF. (A) Immunoblots of RIPA extracts of cells that were grown to confluence in regular medium (UT) or in medium containing 100 pg/ml TNF-α, 50 ng/ml IL-1β or 100 ng/ml IL-6. The extracts were slot blotted to PVDF membranes which were probed with mAb 82E1. The error bars show the means ±SD. (B, C) Low magnification immunofluorescence images of confluent cultures of cells that were grown to confluence in regular medium (B, UT = untreated) or in medium containing 100 ng/ml IL-6 (C). Staining was performed with mAb 82E1 (for the N-terminal of β-CTF/Aβ, green), pAb A8717 (for the C-terminal of APP/β-CTF, red), and DAPI (for nuclei, blue). Exposure time was the same in all cases. Bar, 20 µm.

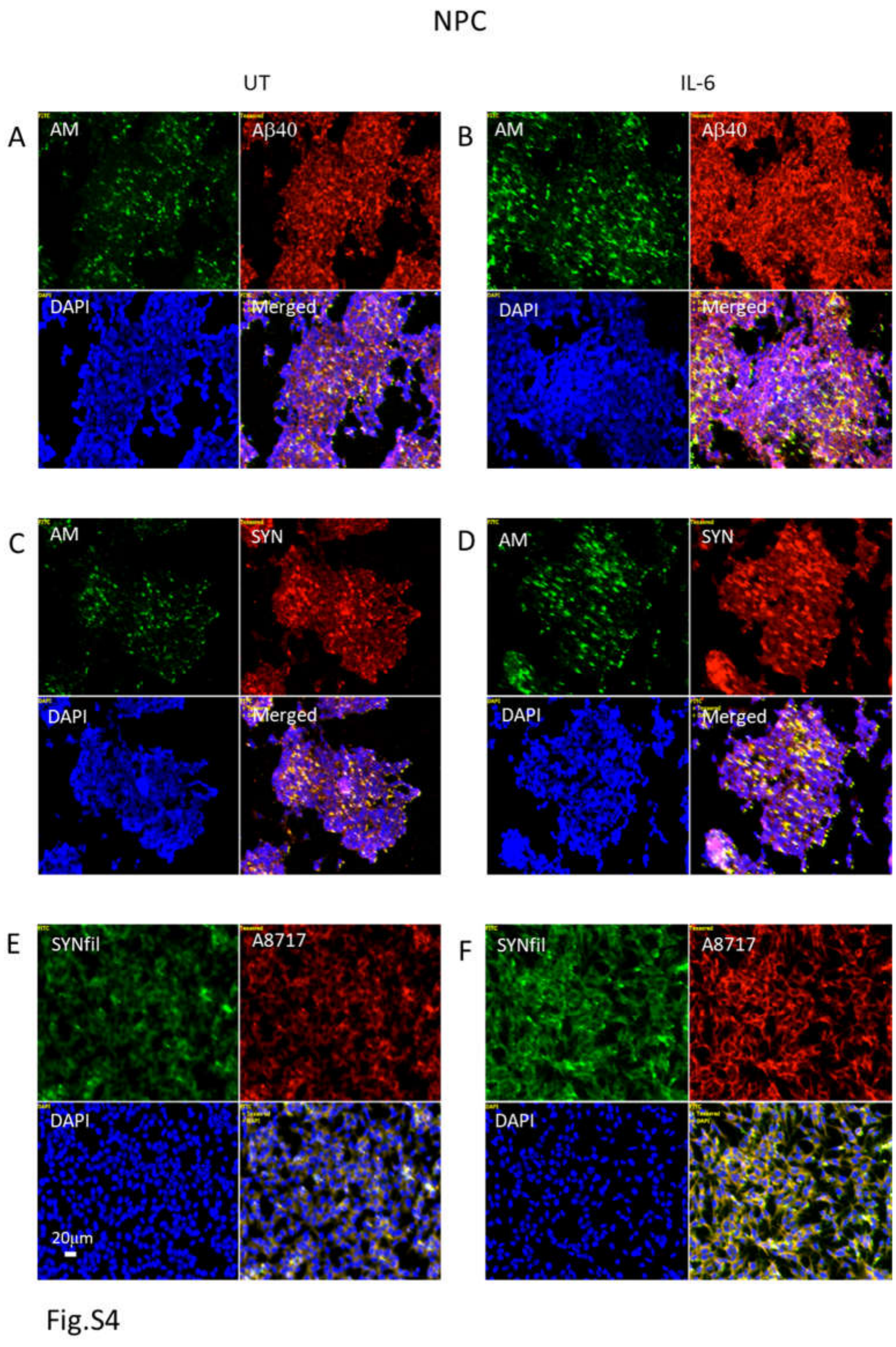

Fig. S4. IL-6 induces increased formation of HS-anMan and SYNfil. Low magnification immunofluorescence images of cells that were grown to confluence in regular medium (A, C, E, UT = untreated) or in medium containing 100 ng/ml IL-6 (B, D, F). Staining was performed with mAb AM (for HS-anMan, green), mAb SYNfil, green), pAb Aβ40 (for the Aβ region of APP/β-CTF, red), pAb SYN (red), pAb A8717 (for the C-terminal of APP/β-CTF, red) and DAPI (for nuclei, blue). Exposure time was the same in all cases. Bar, 20 µm.

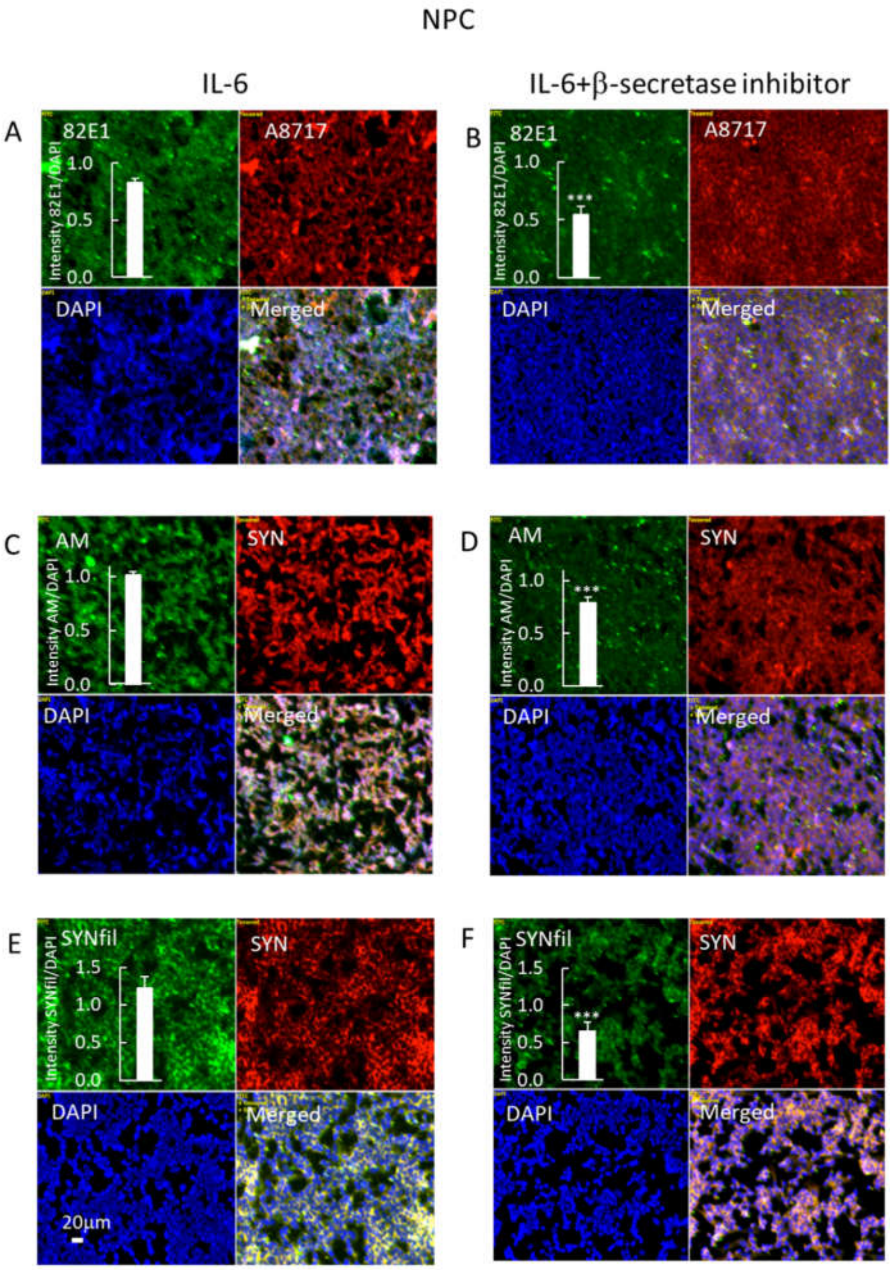

Fig. S5

Fig. S5. Inhibition of β-secretase suppresses IL-6-induced formation of β-CTF, HS-anMan and SYNfil. Low magnification immunofluorescence images of cells that were grown to confluence in medium containing 100 ng/ml IL-6 (A, C, E) or in medium containing 100 ng/ml IL-6 and 100 nM β-inhibitor (LY2811376) (B, D, F: IL-6+β-secretase inhibitor). Staining was performed with mAb 82E1 (for the N-terminal of β-CTF/Aβ, green), mAb AM (for HS-anMan, green), mAb SYNfil (green), pAb A8717 (for the C-terminal of APP/β-CTF, red), pAb SYN (red), and DAPI (for nuclei, blue). The intensities of 82E1, AM, SYNfil and DAPI staining were measured and expressed as the ratios 82E1/DAPI, AM/DAPI and SYNfil/DAPI, respectively, see insets in A-F. The error bars show the means ±SD. Exposure time was the same in all cases. Bar, 20 µm.

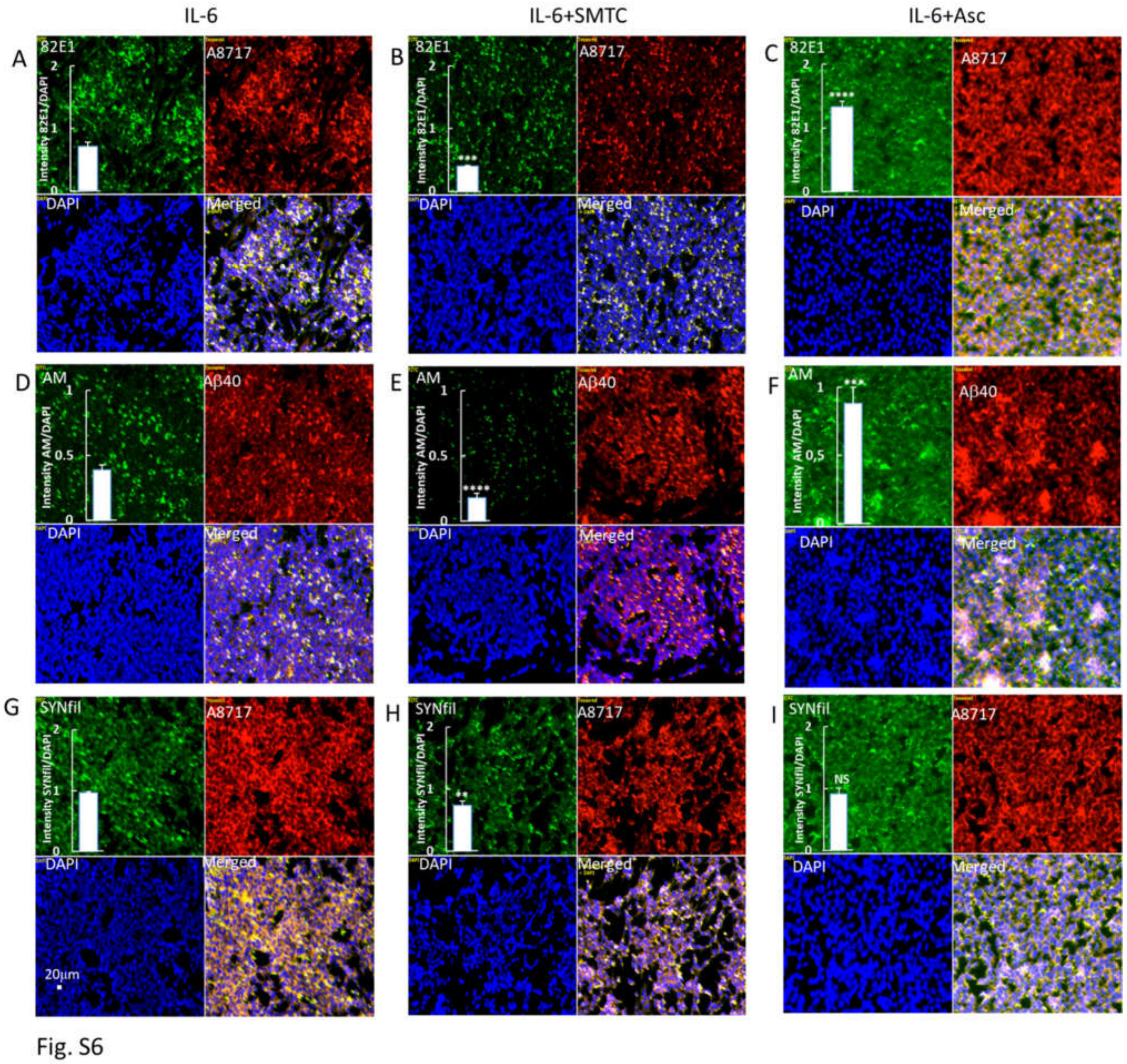

Fig. S6. NO-deprivation suppresses and ascorbate stimulates APP processing and HS-anMan formation in IL-6-treated cells. Low magnification immunofluorescence images of cells that were grown to confluence in medium containing 100 ng/ml IL-6 (A, D, G) or in medium containing 100 ng/ml IL-6 and 100 μM S-methyl-L-thiocitrulline (SMTC) (B, E, H) or in medium containing 100 ng/ml IL-6 and 1 mM ascorbate (C, F, I) (Asc). Staining was performed with mAb 82E1 (for the N-terminal of β-CTF/Aβ, green), mAb AM (for HS-anMan, green), mAb SYNfil (green), pAb A8717 (for the C-terminal of APP/β-CTF, red), pAb Aβ40 (for the Aβ region, red), and DAPI (for nuclei, blue). The intensities of 82E1, AM, SYNfil and DAPI staining were measured and expressed as the ratios 82E1/DAPI, AM/DAPI and SYNfil/DAPI, respectively, see insets in A-I. The error bars show the means ±SD. Exposure time was the same in all cases. Bar, 20 μm.

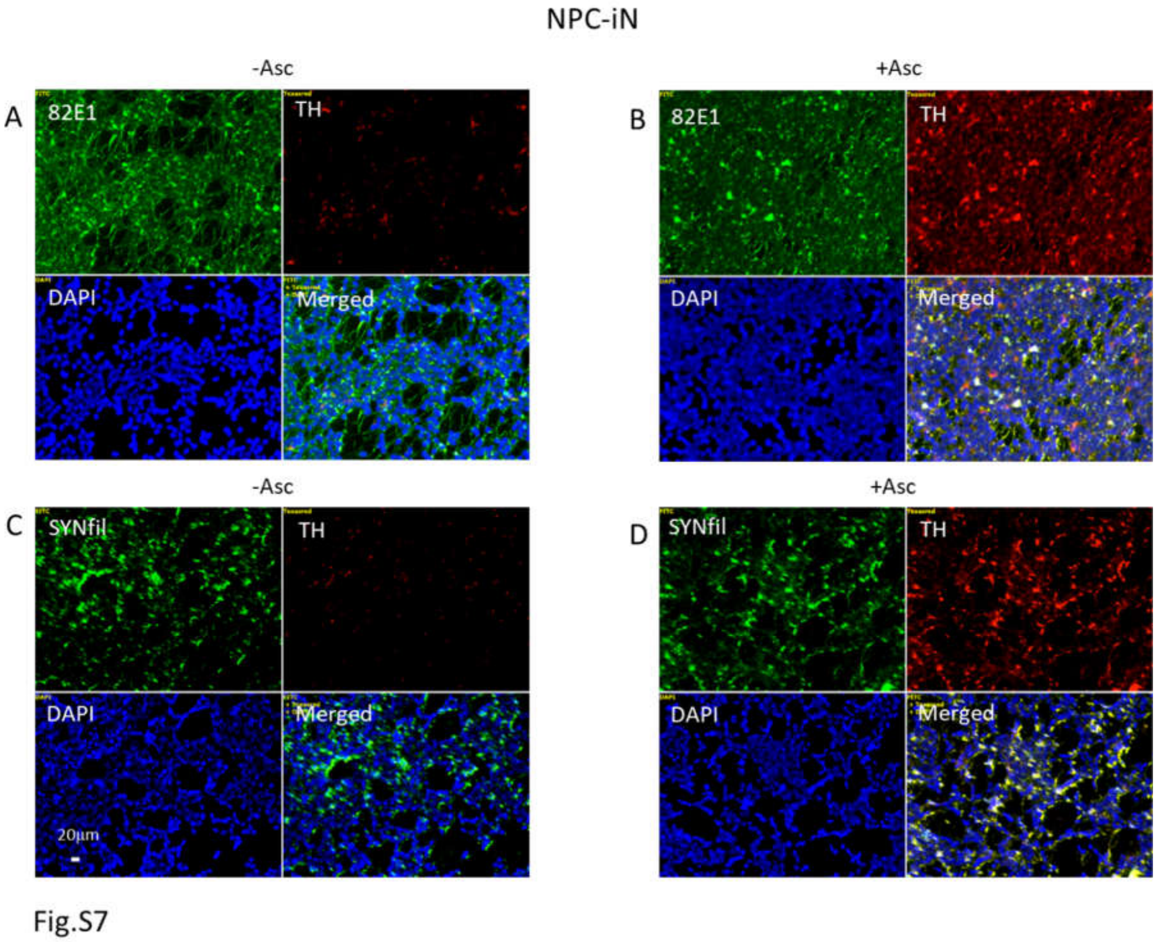

Fig. S7. Differentiation of NPC to NPC-iN requires the presence of ascorbate in the medium. Low magnification immunofluorescence images of cells that were maintained in differentiation medium without added ascorbate (-Asc, A, C) or with 0.1 μM ascorbate (+Asc, B, D). Staining was performed with mAb 82E1 (for the N-terminal of β-CTF/Aβ, green), mAb SYNfil (green), pAb anti-TH (red), and DAPI (for nuclei, blue). Exposure time was the same in all cases. Bar, 20 μm.

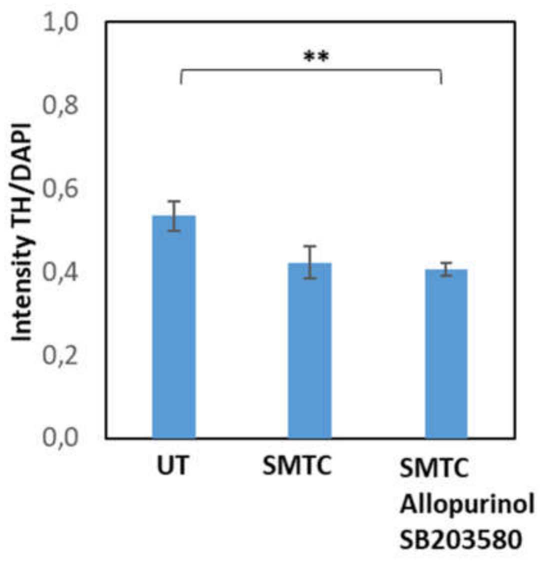

Fig.S8

Fig. S8. Extensive suppression of HS-anMan release from GPC1 during differentiation of NPC to NPC-iN significantly reduces TH staining. The intensities of TH and DAPI staining were measured in low magnification immunofluorescence images of cells that were maintained in complete (+0.1 μM Asc) differentiation medium without further additions (UT = untreated, n=8), or in complete medium containing 100 μM S-methyl-L-thiocitrulline (SMTC, n=6) or in complete medium containing 100 μM S-methyl-L-thiocitrulline (SMTC), 150 μM allopurinol and 1 μM SB203580 (n=6) and expressed as TH/DAPI. The error bars show the means ±SE.
